# Supplementary material for: A deep neural network approach to predicting clinical outcomes of neuroblastoma patients
Source: BMC Med Genomics. 2019 Dec 20;12(Suppl 8):178. doi: 10.1186/s12920-019-0628-y (PMC6923884; doi:10.1186/s12920-019-0628-y)
Supplement: Supplementary file 1 — Additional file 1 Supplementary Figures S1-S2. PDF file. [file 12920_2019_628_MOESM1_ESM.pdf]

Additional file 1 for "A deep neural network approach to predict clinical outcomes of Neuroblastoma patients"

Léon-Charles Tranchevent[1], Francisco Azuaje[1], and Jagath C. Rajapakse[2]

[1]Proteome and Genome Research Unit, Department of Oncology, Luxembourg Institute of Health, Luxembourg.
[2]Bioinformatics Research Center, School of Computer Engineering, Nanyang Technological University, Singapore

List of Figures

S1 Performance of the DNN models with various architectures. 1
S2 Detailed performance of the models trained with different parameters. 2

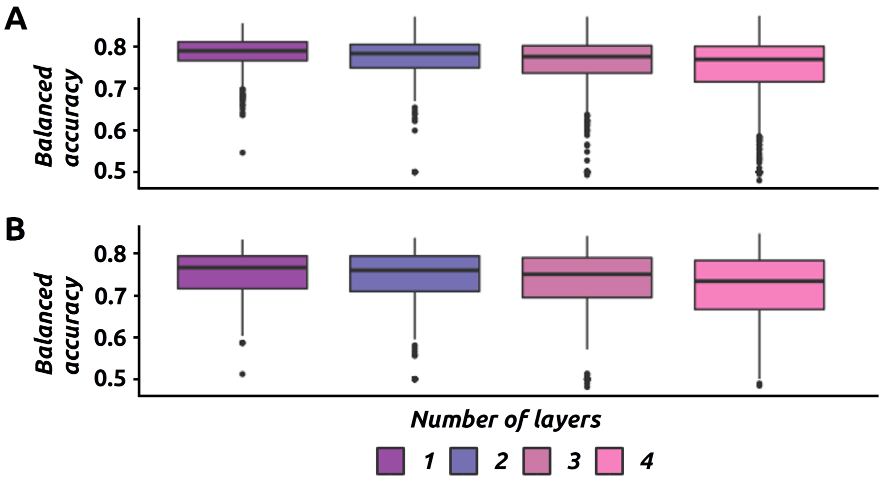

Supplementary Figure S1: The performance (*i.e.*, balanced accuracy) of DNN models with different architectures (*i.e.*, number of layers) for '*Death from disease*' (A) and '*Disease progression*' (B).

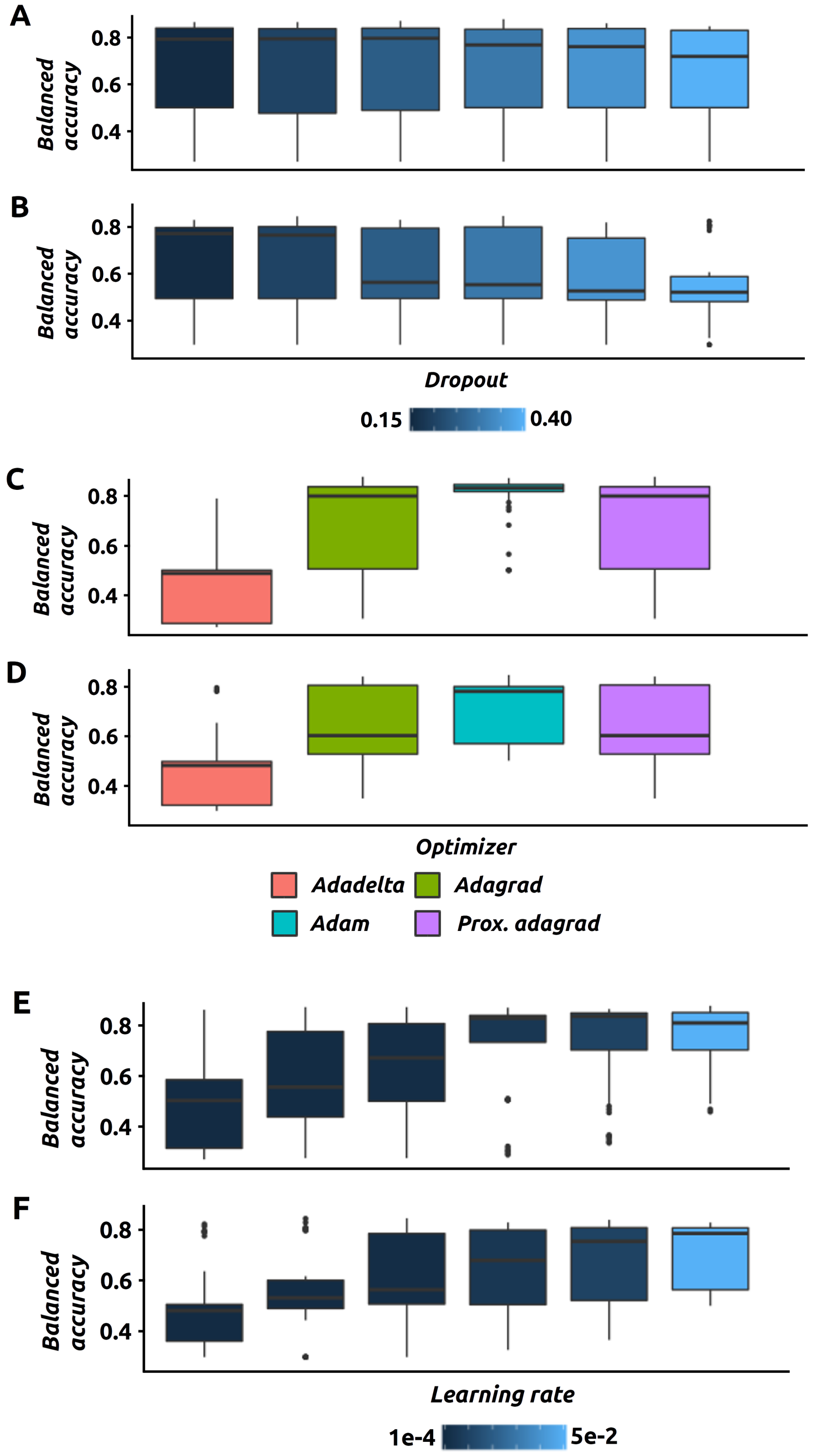

Supplementary Figure S2: The performance (*i.e.*, balanced accuracy) of DNN models in function of hyper-parameter such as dropout (A and B), optimizing strategy (C and D) and learning rate (E and F). Results are displayed for '*Death from disease*' (A, C and E) and '*Disease progression*' (B, D and F).
